# Water and Nitrogen Use Strategies and Their Influencing Mechanisms in Typical Desert Shrubs of the Qaidam Basin, Qinghai–Tibet Plateau, China

**DOI:** 10.3390/plants14243828

**Published:** 2025-12-16

**Authors:** Yunhao Zhao, Hui Chen

**Affiliations:** 1 Hebei Key Laboratory of Environmental Change and Ecological Construction, Hebei Technology Innovation Center for Remote Sensing Identification of Environmental Change, School of Geographical Sciences, Hebei Normal University, Shijiazhuang 050024, China; zhaoyunhao0626@igsnrr.ac.cn; 2 Key Laboratory of Water Cycle and Related Land Surface Processes, Institute of Geographic Sciences and Natural Resources Research, Chinese Academy of Sciences, Beijing 100101, China; 3 College of Resources and Environment, University of Chinese Academy of Sciences, Beijing 100049, China

**Keywords:** Qaidam Basin, desert shrub, *δ*^13^C, *δ*^15^N, plant functional traits, leaf economic spectrum, drought stress

## Abstract

Desert plants develop unique functional traits and resource utilization strategies under environmental stress, among which, water and nitrogen utilization strategies are the key resource utilization strategies for desert plants. Research on plant water and nitrogen utilization and leaf functional traits has rarely involved high-altitude desert shrubs. The synergistic or trade-off relationship between water and nitrogen utilization in desert shrubs remains unclear, and the variation patterns of leaf functional trait combinations related to water and nitrogen utilization along environmental gradients urgently need to be studied. This study takes the typical desert shrubs in the eastern part of the Qaidam Basin on the Qinghai–Tibet Plateau in China as the research object, selects the stable carbon and nitrogen isotopes (*δ*^13^C, *δ*^15^N) of plant leaves to characterize the water use efficiency (WUE) and nitrogen use strategy (NUE) of plants, explores the main leaf functional traits related to water and nitrogen utilization, and analyzes the relationship between leaf functional traits and environmental factors. The results show that the resource utilization traits of desert shrubs can be divided into two groups: water and carbon utilization centered on *δ*^13^C and nutrient utilization centered on *δ*^15^N. There are synergistic or trade-off relationships among plant functional traits. There is a trade-off relationship between water and nitrogen utilization in plants. The leaf functional traits related to water and nitrogen utilization in plants form a “water and nitrogen utilization leaf economic spectrum” along the gradients of temperature, drought, salinity, and nutrients. In conclusion, desert plants adapt to the environment of high cold, drought, high salt content, and limited nutrients by adjusting the relevant leaf functional traits. This study combines the stable carbon and nitrogen isotopes of plant leaves with the combined characteristics of leaf functional traits under different environmental gradients, providing a new perspective for understanding the water and nitrogen utilization strategies of high-altitude desert shrubs and their adaptation mechanisms to arid environments.

## 1. Introduction

Desert ecosystems at high elevations are both ecologically fragile and highly responsive to shifts in global climate. These characteristics render them particularly valuable for investigating how climate change influences ecological processes [1,2]. Exploring the relationship between plant functional traits and resource utilization strategies in desert regions, and their adaptive characteristics to arid environments, is an important aspect of revealing the response of desert regions in the context of global climate change [3]. Desert plant growth is easily limited by water and nitrogen resources, and water and nitrogen utilization strategies are important parameters for understanding the interrelationships between carbon, nitrogen, and water, reflecting the response and adaptation of desert plants to water and nitrogen stresses [4].

The leaf, as the main organ of plant photosynthesis and transpiration, assumes the function of plant energy production and conversion [5]. Leaf functional traits are sensitive to climate change, reflecting the plant’s ability to highly adapt to the environment and self-regulate under complex habitats [6]. Leaf carbon isotopes (*δ*^13^C) are often used to portray plant water–carbon balance and are a surrogate for assessing the long-term water use efficiency of plants, with higher *δ*^13^C values indicating higher water use efficiency [7]. Leaf nitrogen isotopes (*δ*^15^N) are often used to portray plant nitrogen fixation pathways and the amount of nitrogen fixation, which is a useful tool for studying the physiology of plant nitrogen assimilation and nitrogen use efficiency [8]. It can indicate the effectiveness (availability) of soil nitrogen, reflecting the nitrogen cycle of plants, nitrogen uptake and utilization, and the rate of nitrogen conversion and loss. The higher *δ*^15^N indicates the higher nitrogen utilization efficiency of plants [9]. Therefore, plant leaf *δ*^13^C and *δ*^15^N are commonly used as surrogate indicators of plant water and nitrogen utilization strategies [10].

In drought-stressed environments, plant growth is jointly limited by nitrogen and water availability, and there is a trade-off between plant nitrogen use efficiency (NUE) and water use efficiency (WUE) [11]. Conesa et al. [12] found that there is a constraint between plant water use efficiency and nitrogen use efficiency, that plants are not able to optimally utilize water and nitrogen at the same time, and that this equilibrium process is disturbed by climate and soil nutrients [13]. Zhao et al. [14] indicated that the relationship between carbon and nitrogen isotopes varied with plant species and photosynthetic pathways, with a significant negative correlation between leaf *δ*^13^C and *δ*^15^N in C_3_ plants, and a significant positive correlation between leaf *δ*^13^C and *δ*^15^N in leguminous and C_4_ plants, suggesting that there is a trade-off or a synergistic relationship between the strategies of water and nitrogen utilization. Gong et al. [15] reported that, under conditions of nitrogen enrichment, *Leymus chinensis* lowers its NUE while increasing WUE. This adjustment suggests that the balance between NUE and WUE enables plants to optimize the utilization of both nitrogen and water resources. Nevertheless, an excessive nitrogen supply can elevate leaf nitrogen concentration and the N:P ratio, intensifying phosphorus limitation. Such changes may suppress photosynthetic performance and disrupt the trade-off between NUE and WUE [16]. Consequently, examining the interaction between NUE and WUE across different plant functional groups is essential for understanding their adaptive strategies to environmental variation and for clarifying patterns of community development.

The trade-off between water and nitrogen use efficiency is primarily governed by the physiological characteristics of plant leaves. Nitrogen influences photosynthetic activity, while water availability regulates transpiration, and both processes are jointly controlled by stomatal behavior [17]. An open stomatal structure, which helps to increase photosynthesis, also leads to greater water loss [18]. A reduction in stomatal conductance can enhance plant water use efficiency (WUE) under drought or water-limited conditions, but this improvement often comes at the cost of lower net photosynthetic rates and diminished nitrogen use efficiency (NUE) [19]. Hu et al. [11] found that, under water-constrained conditions, plants often maintain a carbon budget mechanism by increasing WUE (at the cost of reducing NUE). Shifts in nutrient availability further influence both WUE and NUE, as well as the balance between them. For instance, nitrogen enrichment has been shown to reduce both NUE and WUE, indicating that soil nitrogen and water imposed weaker constraints on plant growth than other resources (e.g., phosphorus or light) [11]. Moreover, Crous et al. [20] reported a positive association between leaf phosphorus concentration, WUE, and photosynthetic capacity, noting that many species experiencing phosphorus limitation also display reduced NUE [21]. Environmental changes such as temperature and precipitation also affect *δ*^13^C and *δ*^15^N in dryland plants. Niu et al. [22] found that soil salinity, water conditions (soil moisture and precipitation), and temperature were the key drivers of *δ*^13^C and *δ*^15^N. Therefore, plant strategies for water and nitrogen use are likely shaped by multiple interacting factors, making it particularly important to investigate how leaf functional traits and environmental conditions jointly influence water and nitrogen utilization.

Current research on the environmental responses of leaf functional traits and resource utilization strategies has primarily focused on trees and herbaceous plants in global and ecologically favorable regions [23,24], with limited studies on desert shrubs in ecologically impoverished areas. Research on plant leaf functional traits and resource utilization strategies under environmental stress requires urgent reinforcement, as the relationships between leaf functional trait combinations and environmental changes, as well as water and nitrogen utilization strategies, remain unclear. Given that high-altitude desert regions face concurrent drought and nitrogen stress, and that plant water and nitrogen use efficiencies may exhibit trade-offs, we hypothesize that, as drought indices increase, shrub leaf water use efficiency (*δ*^13^C) will significantly improve, while nitrogen use efficiency (*δ*^15^N) will significantly decline, exhibiting a negative correlation between the two. Based on this, this study aims to address the following scientific questions: (1) What is the relationship between water and nitrogen use in typical desert shrubs in the eastern Qaidam Basin? (2) Which leaf functional traits influence plant water and nitrogen use? (3) What is the leaf economic spectrum of water and nitrogen use across different environmental gradients? To address these questions, this study employs field surveys, leaf functional trait measurements, regression analysis, and redundancy analysis to systematically examine the coupling mechanisms between leaf functional traits and water/nitrogen use across varying environmental gradients.

## 2. Results

### 2.1. The Relationship Between Water and Nitrogen Utilization in Plants

**Figure 1:**
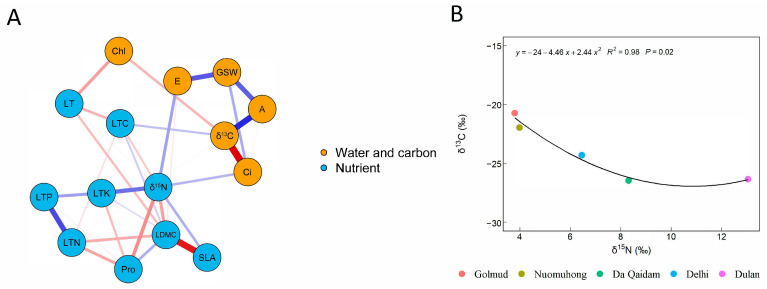
Relationship between plant leaf trait networks (**A**) and water and nitrogen use efficiency (**B**). Note: Plant functional traits included all plant species at sampling sites of leaf stable carbon isotope (*δ*^13^C), stable nitrogen isotope (*δ*^15^N), net photosynthetic rate (A), transpiration rate (E), intercellular carbon dioxide concentration (Ci), stomatal conductivity (GSW), chlorophyll content (Chl), leaf carbon content (LTC), leaf nitrogen content (LTN), leaf phosphorus content (LTP), specific leaf area (SLA), proline content (Pro), dry matter content of leaves (LDMC), leaf thickness (LT). Among them, leaf water content (WC) has strong collinearity with other variables, so they are excluded. The blue and red lines represent positive correlation and negative correlation, respectively. The thicker the line, the stronger the correlation.

Network analysis showed that the leaf functional traits of desert shrubs in the eastern Qaidam Basin can be clearly divided into two groups (Figure 1A). One group is centered on leaf *δ*^13^C and comprises water–carbon use-related traits associated with gas exchange and photosynthetic processes, including net photosynthetic rate (A), transpiration rate (E), stomatal conductance (GSW), intercellular carbon dioxide concentration (Ci), and chlorophyll content (Chl). The other group is centered on leaf *δ*^15^N and comprises nutrient use-related traits, including leaf nitrogen content (LTN), leaf phosphorus content (LTP), leaf dry matter content of leaves (LDMC), specific leaf area (SLA), leaf thickness (LT), proline content (Pro), leaf carbon content (LTC), and total leaf potassium (LTK).

The trait sets entered into the network analysis were assigned according to the commonly used classification of leaf functional traits in previous studies [12,25,26,27,28]. The analysis further confirmed that plant functional traits can be divided into these two groups and revealed trade-offs or synergies among individual traits. Bivariate regression indicated a significant non-linear relationship between leaf *δ*^13^C and *δ*^15^N (*p* < 0.05, Figure 1B). Thus, a trade-off exists between water and nitrogen use in desert shrubs of the eastern Qaidam Basin.

### 2.2. δ^13^C, δ^15^N, and Related Leaf Functional Traits

**Figure 2:**
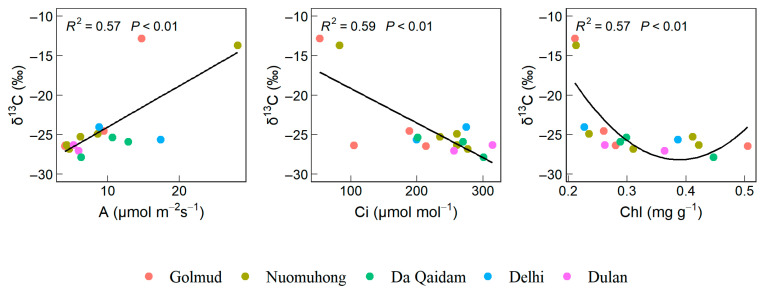
Leaf functional traits significantly correlated with *δ*^13^C of leaves. Note: Stable carbon isotope (*δ*^13^C), net photosynthetic rate (A), intercellular carbon dioxide concentration (Ci), chlorophyll content (Chl).

Bivariate regression analysis revealed that leaf *δ*^13^C exhibited a significant positive correlation with A, a significant negative correlation with Ci, and a nonlinear association with Chl, characterized by an initial decline followed by an increase (Figure 2).

**Figure 3:**
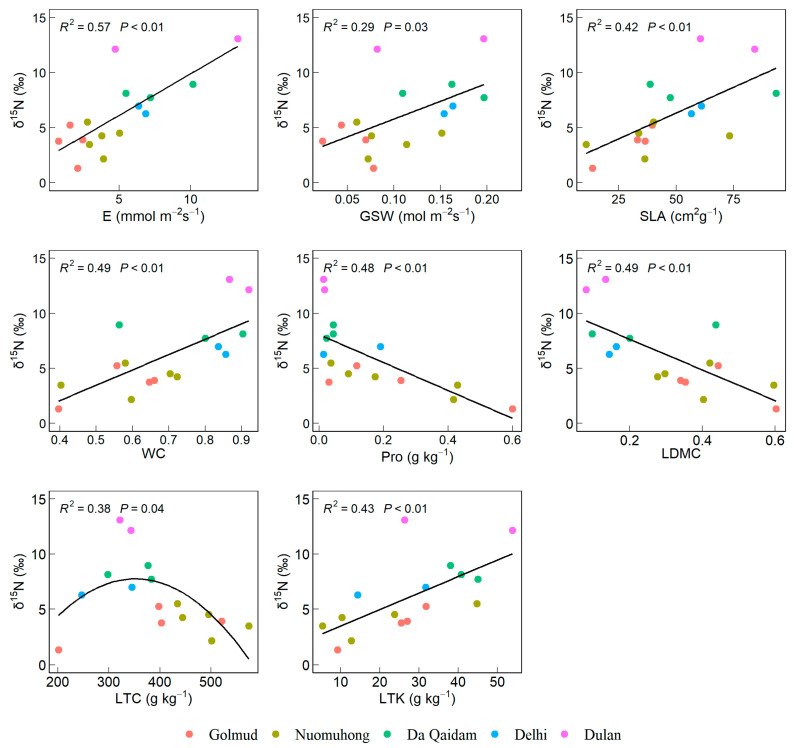
Leaf functional traits significantly correlated with *δ*^15^N of leaves. Note: Stable nitrogen isotope (*δ*^15^N), transpiration rate (E), stomatal conductivity (GSW), specific leaf area (SLA), leaf water content (WC), proline content (Pro), dry matter content of leaves (LDMC), leaf carbon content (LTC), leaf total potassium (LTK).

Leaf *δ*^15^N exhibited significant positive correlations with E, GSW, SLA, WC, and LTK, significant negative correlations with Pro and LDMC, and a non-linear relationship with LTC characterized by an initial increase followed by a decrease (*p* < 0.05, Figure 3).

**Figure 4:**
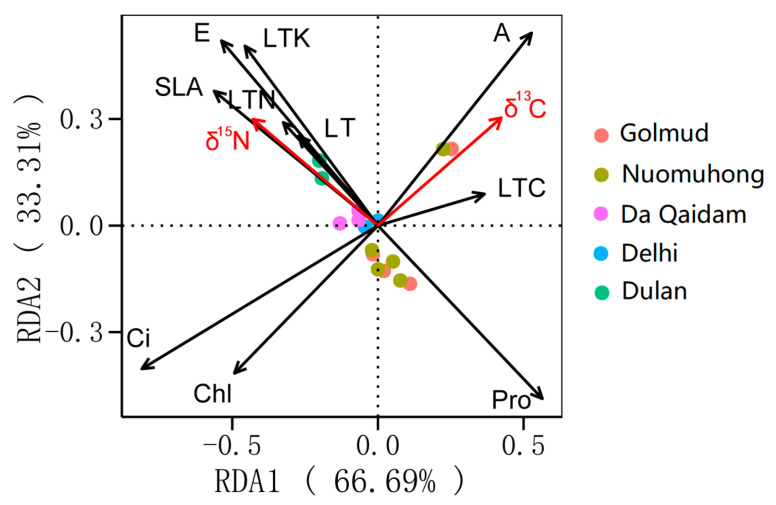
RDA of *δ*^13^C, *δ*^15^N and related leaf functional traits. Note: Stable carbon isotope (*δ*^13^C), stable nitrogen isotope (*δ*^15^N), net photosynthetic rate (A), transpiration rate (E), intercellular carbon dioxide concentration (Ci), chlorophyll content (Chl), leaf nitrogen content (LTN), specific leaf area (SLA), proline content (Pro), leaf thickness (LT), leaf carbon content (LTC), leaf total potassium (LTK).

To further examine the associations between *δ*^13^C, *δ*^15^N, and leaf functional traits, RDA was conducted to identify characteristics related to the coupled utilization of water and nitrogen. The analysis showed that leaf traits together accounted for 68.6% of the total variation in *δ*^13^C and *δ*^15^N. The constrained eigenvalues of the first and second axes were 66.69% and 33.31%, respectively, explaining the full variance, with the first axis providing the dominant contribution (Figure 4). Excluding the collinear leaf functional traits, hierarchical segmentation (HP) was conducted on net photosynthetic rate (A), transpiration rate (E), intercellular carbon dioxide concentration (Ci), chlorophyll content (Chl), leaf nitrogen content (LTN), specific leaf area (SLA), proline content (Pro), leaf thickness (LT), leaf carbon content (LTC), and leaf total potassium (LTK). The contribution of leaf functional traits was analyzed, and the importance ranking of each factor was obtained: Ci (28.06%, *p* < 0.01), A (17.4%, *p* < 0.05), Chl (12.06%, *p* < 0.05), E (11.53%, *p* < 0.05), Pro (7.14%, *p* < 0.05), LTK (7%, *p* < 0.05), SLA (5.38%, *p* < 0.05), LTC (4.45%, *p* > 0.05), LT (4.23%, *p* > 0.05), and LTN (2.7%, *p* > 0.05), indicating that Ci, A, Chl, E, Pro, LTK, and SLA are key leaf functional traits influencing *δ*^13^C and *δ*^15^N.

### 2.3. Relationships Between Leaf Functional Traits Related to Water and Nitrogen Use and Environmental Factors

Based on the above analysis, plant functional traits related to water utilization include the following: leaf stable carbon isotope (*δ*^13^C), net photosynthetic rate (A), intercellular carbon dioxide concentration (Ci), chlorophyll content (Chl). Traits associated with nitrogen utilization include the following: leaf stable nitrogen isotope (*δ*^15^N), transpiration rate (E), stomatal conductivity (GSW), leaf water content (WC), dry matter content of leaves (LDMC), leaf nitrogen content (LTN), specific leaf area (SLA), proline content (Pro), leaf carbon content (LTC), leaf total potassium (LTK). Through redundancy analysis (RDA), the key traits influencing coupled water–nitrogen utilization were screened as follows: *δ*^13^C, *δ*^15^N, Ci, A, Chl, E, Pro, LTK, and SLA.

**Figure 5:**
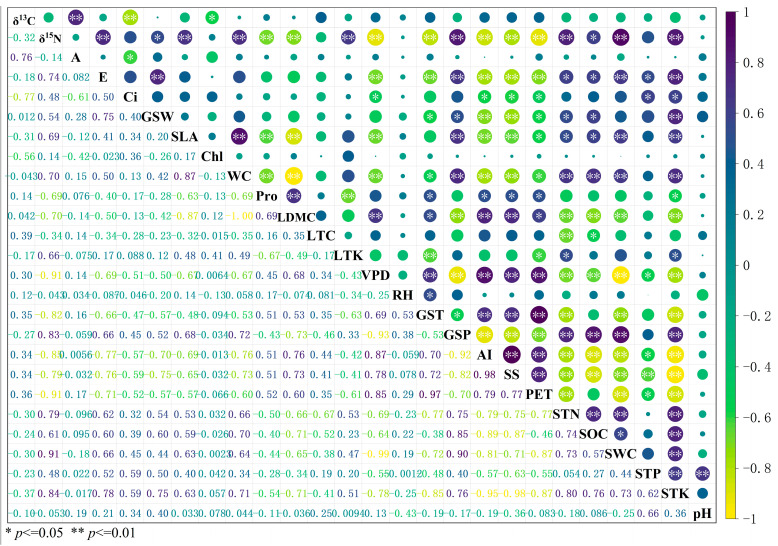
Relationships between leaf functional traits associated with water and nitrogen use and key environmental factors. Note: Plant functional traits include leaf stable carbon isotope (*δ*^13^C), stable nitrogen isotope (*δ*^15^N), net photosynthetic rate (A), transpiration rate (E), intercellular carbon dioxide concentration (Ci), stomatal conductivity (GSW), chlorophyll content (Chl), leaf water content (WC), dry matter content of leaves (LDMC), leaf nitrogen content (LTN), specific leaf area (SLA), proline content (Pro), leaf thickness (LT), leaf carbon content (LTC), leaf total potassium (LTK). Climate factors include water vapor pressure difference (VPD), relative humidity (RH), growing season temperature (GST), growing season precipitation (GSP), annual aridity index (AI), potential evapotranspiration (PET). Soil factors include soluble total salt content (SS), soil total phosphorus (STP), soil total potassium (STK), soil total nitrogen (STN), soil water content (SWC), soil organic carbon (SOC), and soil pH.

Among the environmental factors affecting water- and nitrogen-related leaf traits, growing season temperature (GST), annual aridity index (AI), potential evapotranspiration (PET), soluble total salt (SS) content, soil potassium (STK), and soil total nitrogen (STN) significantly influenced most traits (*δ*^15^N, E, GSW, SLA, WC, Pro, LDMC) (Figure 5). PET and AI yielded similar results, both indicating environmental moisture conditions with strong collinearity. As the AI algorithm integrates potential evapotranspiration and annual precipitation, it is more representative; thus, AI was selected for subsequent analysis. Among soil factors, STK and STN yielded similar results with strong collinearity. Since soil total nitrogen (STN) serves as the primary nutrient-limiting factor in the desert soils of the Qaidam Basin, it was chosen as the key variable for subsequent analyses. Ultimately, four primary environmental factor gradients—GST, AI, SS, and STN (temperature, aridity, salinity, nutrients)—were chosen for further investigation.

#### 2.3.1. The Gradient Changes in Leaf Functional Traits Related to Water Use Along the Main Environmental Factors

**Figure 6:**
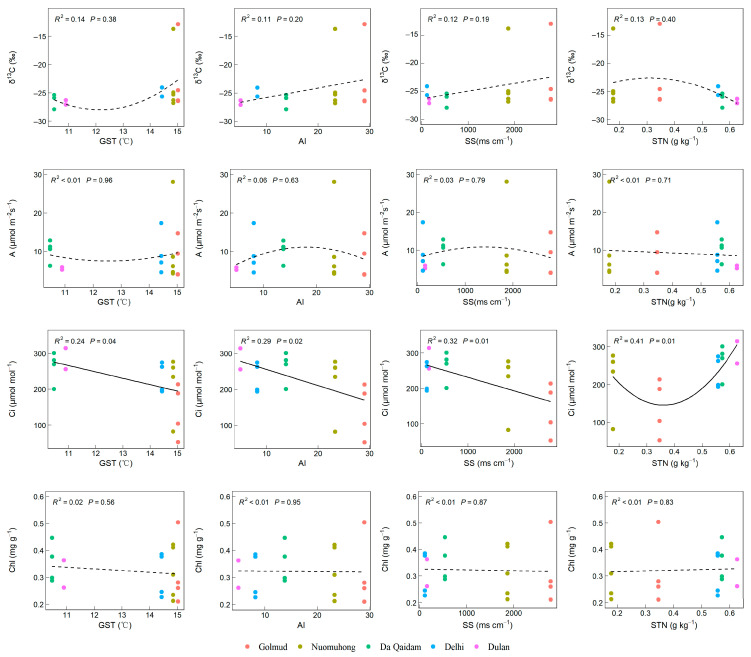
The relationship between leaf functional traits related to water use and environmental factors. Note: Stable carbon isotope (*δ*^13^C), net photosynthetic rate (A), intercellular carbon dioxide concentration (Ci), chlorophyll content (Chl), growing season temperature (GST), annual aridity index (AI), soluble total salt (SS) content, soil total nitrogen (STN). The solid lines in the figure represent significant trends (*p* < 0.05), dotted lines represent non-significant trends (*p* > 0.05).

Leaf stable carbon isotope (*δ*^13^C) showed an upward trend with increasing GST and exhibited a positive correlation with AI and SS, though the relationship was not statistically significant (*p* > 0.05, Figure 6). The leaf intercellular carbon dioxide concentration (Ci) was significantly negatively correlated with GST, AI, and SS, and showed a nonlinear pattern—initially decreasing and then increasing—with rising STN levels (*p* < 0.05). The net photosynthetic rate (A) and chlorophyll content (Chl) showed no significant relationship with GST, AI, SS, or STN (*p* > 0.05, Figure 6). Consequently, among leaf functional traits related to plant water use, Ci demonstrated significant variation along the GST, AI, SS, and STN gradient axes.

#### 2.3.2. The Gradient Changes in Leaf Functional Traits Related to Nitrogen Use Along the Main Environmental Factors

**Figure 7:**
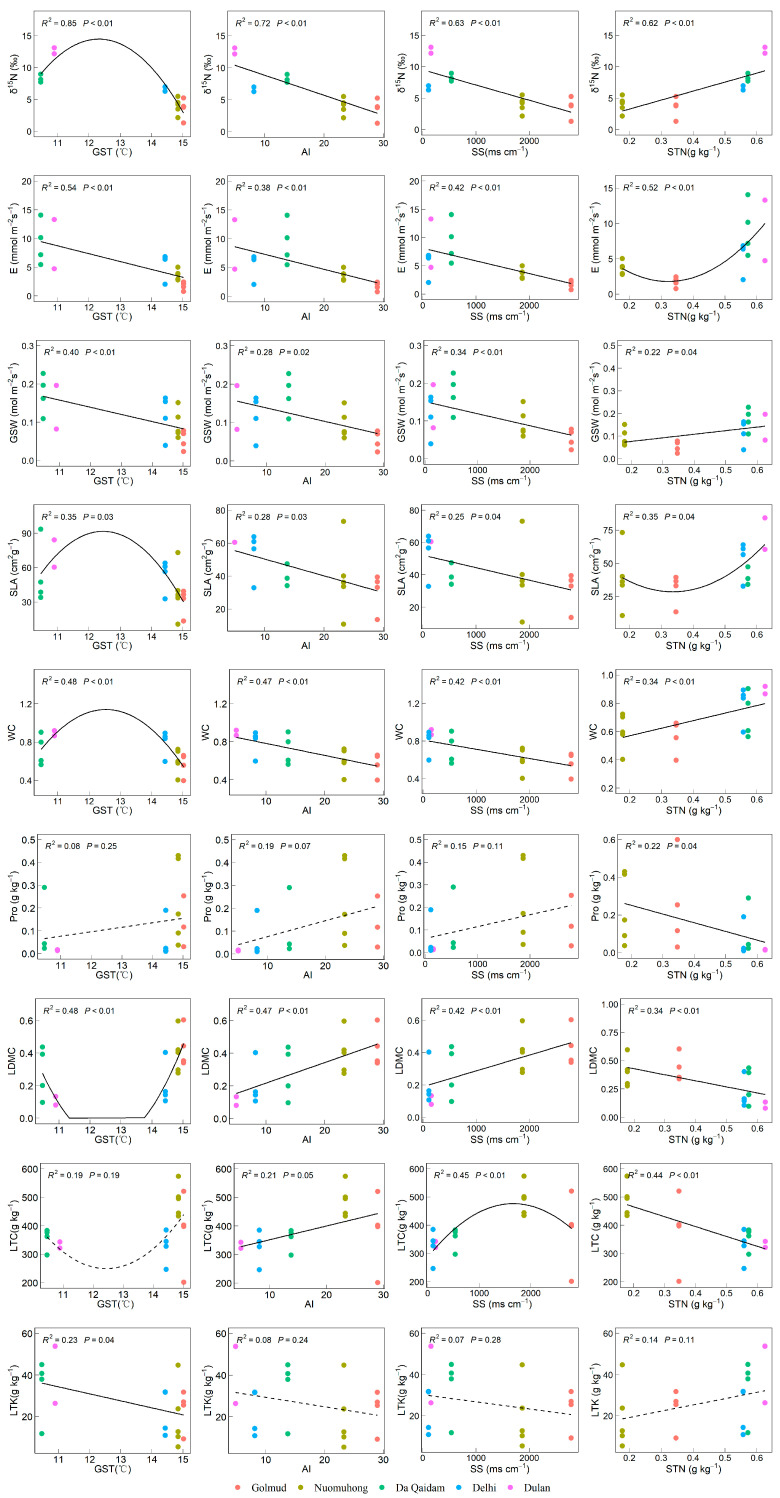
The relationship between leaf functional traits related to nitrogen use and environmental factors. Note: Stable nitrogen isotope (*δ*^15^N), transpiration rate (E), stomatal conductivity (GSW), specific leaf area (SLA), leaf water content (WC), dry matter content of leaves (LDMC), proline content (Pro), leaf carbon content (LTC), leaf total potassium (LTK), growing season temperature (GST), annual aridity index (AI), soluble total salt (SS) content, soil total nitrogen (STN). The solid lines in the figure represent significant trends (*p* < 0.05), dotted lines represent non-significant trends (*p* > 0.05).

Among the leaf traits related to nitrogen utilization, *δ*^15^N increased with GST before declining, showed significant negative correlations with AI and SS, and a positive one with STN (*p* < 0.05). The transpiration rate (E) and stomatal conductance (GSW) were both negatively correlated with GST, AI, and SS. However, E exhibited a unimodal pattern with increasing STN, first decreasing and then rising, whereas GSW showed a significant positive correlation with STN (*p* < 0.05). The specific leaf area (SLA) and leaf water content (WC) were significantly negatively correlated with AI and SS, and showed a trend of first increasing and then decreasing with GST, with an upward trend as STN increased. Proline (Pro) was negatively correlated with STN but showed non-significant positive associations with GST, AI, and SS. Leaf dry matter content (LDMC) was positively related to AI and SS and negatively to STN, while leaf total carbon (LTC) was positively correlated with AI, varied nonlinearly with SS, and decreased with STN. Leaf total potassium (LTK) was negatively correlated with GST and weakly related to the other factors. Overall, *δ*^15^N, E, GSW, SLA, WC, and LDMC showed significant variation along the GST, AI, SS, and STN gradients (Figure 7).

#### 2.3.3. The Gradient Changes in Leaf Functional Traits Related to Water–Nitrogen Coupling Utilization Along the Main Environmental Factors

According to the results of 2.2 RDA, the functional traits of plant leaves that affect the coupling utilization of water and nitrogen include leaf stable carbon isotope (δ^13^C), stable nitrogen isotope (δ^15^N), net photosynthetic rate (A), transpiration rate (E), intercellular carbon dioxide concentration (Ci), chlorophyll content (Chl), specific leaf area (SLA), proline content (Pro), and leaf total potassium (LTK) (Figure 4). Among them, δ^13^C, Ci, δ^15^N, E, Pro, LTK, and SLA show regular changes along the gradients of growing season temperature (GST), annual aridity index (AI), soluble total salt content (SS), and soil total nitrogen (STN). The δ^13^C and Pro increase along the GST gradient, δ^15^N and SLA increase first and then decrease, and Ci, E, and LTK decrease. Along the AI and SS gradients, δ^13^C and Pro increase, while Ci, δ^15^N, E, LTK, and SLA decrease. Along the STN gradient, Pro decreases, Ci, E, and SLA decrease first and then increase, and δ^15^N and LTK increase (Figure 6 and Figure 7).

## 3. Discussion

### 3.1. The Relationship Between Plant Water and Nitrogen Use

Leaf *δ*^13^C provides a reliable proxy for long-term water use efficiency (WUE), with higher *δ*^13^C values indicating greater water use efficiency [7]. Gas exchange parameters (net photosynthetic rate, transpiration rate, intercellular carbon dioxide concentration, stomatal conductivity) reflect instantaneous or short-term physiological states. These parameters interact directly and collectively influence *δ*^13^C [26]. Chl correlates with photosynthetic potential and undergoes adjustments in arid environments to adapt to variations in photosynthetic assimilation rates and water availability [27,28]. Therefore, the above represents water and carbon utilization traits centered on *δ*^13^C in plant leaves (Figure 1A).

In the arid and nutrient-poor environment of the eastern Qaidam Basin, plants tend to adopt a conservative “slow investment-return” strategy. Leaf *δ*^15^N values reflect nitrogen sources and utilization efficiency, serving as key indicators for elucidating plant nitrogen utilization strategies [25]. Leaf phosphorus and nitrogen content (LTP, LTN), along with their ratios (e.g., N:P), reflect plant nutrient status and utilization efficiency [12]. Leaf carbon content (LTC) and structural traits (dry matter content of leaves and specific leaf area and leaf thickness) correlate with nutrient utilization and recycling efficiency [29]. Proline content (Pro) and leaf total potassium (LTK) serve as osmotic regulators accumulated by plants under abiotic stresses such as drought and salinity, influenced by overall nutrient uptake and allocation strategies [30]. Therefore, the above are the nutrient utilization traits centered on *δ*^15^N in plant leaves (Figure 1A).

Water and nitrogen represent the two most essential resources for plant growth. Extended periods of drought greatly hinder nitrogen mobility in arid soils, leading to frequent co-limitations of water and nitrogen in the Qaidam Basin [31]. Consequently, efficient resource utilization is particularly crucial for plant growth and survival in harsh environments. Extensive studies have shown that plants adjust their resource use strategies in response to variations in resource availability. When one essential resource becomes more limited, plants often increase their utilization efficiency for that resource, potentially at the cost of reduced efficiency in using another, less limiting resource [19]. This study reveals a trade-off between water and nitrogen utilization strategies in plants within arid ecosystems experiencing concurrent nutrient and water limitations. This finding aligns with Hu et al.’s [11] observation of a significant negative correlation between water use efficiency (WUE) and nitrogen use efficiency (NUE) under arid conditions. As drought severity increases, plants reduce transpiration and specific leaf area to enhance water use efficiency (WUE), while inhibiting nutrient transport to lower nitrogen use efficiency (NUE) [32]. Leaf-level physiological trade-offs result from the tension optimizing of WUE and NUE. This compromise may manifest in C_3_ plants, particularly under water or nitrogen gradients [33,34]. In this study, all plant species at the Qaidam Basin sampling sites were C_3_ plants, except for the *Calligonum korlaensethe*, which is a C_4_ plant. Given the simple structure of desert ecosystems, a trade-off between plant WUE and NUE exists regardless of species or life form: as aridity increases, leaf stomatal conductance decreases and intercellular carbon dioxide concentration declines, leading to improved plant water use efficiency. Concurrently, the efficiency of plant nutrient resource acquisition diminishes, resulting in reduced NUE.

### 3.2. Leaf Functional Traits That Affect Water and Nitrogen Utilization

Leaf functional traits are strongly linked to plant resource use strategies. Desert plants adapt to arid stress environments by altering leaf functional traits to enhance WUE and NUE [35]. A high net photosynthetic rate (A) means that plants can convert CO_2_ into organic matter more quickly, thereby achieving higher growth and production [36]. A high photosynthesis rate or low stomatal conductance often leads to a low internal and external partial pressure ratio of CO_2_ (Ci: Ca), resulting in weak carbon isotope discrimination and thus a higher *δ*^13^C value of plant carbohydrates [26]. Farquhar et al. [37] demonstrated that WUE increases as Ci: Ca decreases. Therefore, plants with a higher photosynthetic rate also have relatively higher water use efficiency. However, high intercellular carbon dioxide concentration hinders light reactions, reducing the photosynthetic rate of plant leaves and preventing plants from effectively utilizing water for photosynthesis, thereby lowering WUE. Therefore, in this study, leaf *δ*^13^C was significantly positively correlated with A and significantly negatively correlated with Ci (Figure 2). Pan et al. [26] found that, as regional temperature and precipitation decrease, plants tend to develop leaf traits associated with higher photosynthetic capacity, such as elevated N concentration, chlorophyll (Chl), and specific leaf area (SLA). This enhances photosynthetic potential, leading to improved water use efficiency. In contrast, our findings in the typical desert shrub study area of the Qaidam Basin show a negative correlation between leaf *δ*^13^C and Chl. This occurs because, under extreme drought conditions, plants may reduce chlorophyll content to lower photosynthetic activity, thereby minimizing water loss and increasing leaf *δ*^13^C. Furthermore, numerous studies indicate that chlorophyll content decreases under drought stress [27,28].

Increased gas-space conductance (GSW) accelerates the transpiration rate (E), which enhances photosynthesis and promotes more efficient utilization of nutrients such as nitrogen and phosphorus during photosynthesis, thereby improving NUE [18]. Potassium is a vital nutrient required for the normal growth and development of plants, serving as a cofactor for multiple enzymes and playing a crucial role in stomatal movement [30]. Deficiencies in these nutrients reduce leaf photosynthetic rates, thereby affecting plant NUE. Plants exhibiting higher nutrient concentrations and a larger specific leaf area (SLA) tend to have greater capacities for photosynthesis and transpiration, resulting in elevated rates of these physiological processes [38]. SLA exhibits a negative correlation with LDMC. As specific leaf area increases, leaf dry matter content decreases [39], while nitrogen allocation per unit mass of photosynthetic organs increases to maintain high photosynthetic capacity. Under drought stress, desert plants exhibit low leaf water content, premature leaf senescence, reduced phloem transport, and diminished photosynthetic capacity, all of which impair nutrient uptake [40]. Concurrently, they produce high levels of proline (Pro) to maintain cellular function and enhance drought tolerance [30]. Consequently, our study revealed that leaf stable nitrogen isotope (*δ*^15^N) in desert shrubs of the Qaidam Basin showed significant positive correlations with transpiration rate (E), stomatal conductivity (GSW), leaf water content (WC), specific leaf area (SLA), and leaf total potassium (LTK), while exhibiting significant negative correlations with Pro and LDMC.

Thus, a synergistic relationship exists between *δ*^13^C and A, and a trade-off relationship exists between *δ*^13^C and Ci and Chl. *δ*^15^N exhibits synergistic relationships with E, GSW, SLA, WC, and LTK, while showing trade-offs with Pro and LDMC. Leaf functional traits influencing integrated water–nitrogen utilization include *δ*^13^C, *δ*^15^N, Ci, A, Chl, E, Pro, LTK, and SLA, with synergistic or trade-off relationships among these traits. Plants enhance water use efficiency by increasing photosynthetic rate and proline content while decreasing transpiration rate, reducing intercellular CO_2_ concentration, specific leaf area, chlorophyll content, and leaf nutrient content. Concurrently, plant nutrient uptake rates decline, inhibiting nutrient transport and reducing nitrogen use efficiency—a result of plant adaptation to stressed growth environments.

### 3.3. Leaf Economic Spectrum of Water and Nitrogen Utilization

In addition to the influence of the intrinsic physiological mechanisms of plants, environmental factors such as climate and soil also have a significant impact on the water and nitrogen utilization strategies of plants. Desert plants are under stress from water (precipitation, humidity, soil moisture content, etc.), salt, and nutrients (soil N, P, etc.) [41,42], thus developing unique resource utilization strategies. Variation in leaf traits along gradients of temperature, precipitation, and soil salinity illustrates the capacity of plants to adapt to changing environmental conditions [43,44].

As humidity increases, the photosynthetic capacity of plants decreases, and stomata become smaller and denser. In hot and humid tropical forests, the reduction in leaf WUE is driven by lower photosynthesis and higher water loss due to increased stomatal conductance [26]. However, in arid regions, the increase in potential evapotranspiration (PET), annual drought index (AI), and saturated vapor pressure (VPD) will enable plants to utilize scarce water resources more effectively [45]. Hu et al. [11] found that plants can increase their WUE by reducing stomatal conductance, decreasing transpiration rate and intercellular carbon dioxide concentration. Dong et al. [46] found that the *Nitraria tangutorum* increased the WUE (leaf *δ*^13^C value) by reducing the leaf area and specific leaf area. *Reaumuria soongorica* adapts to arid environments by increasing the proline content in leaves [30]. Salt stress induces stomatal closure, restricting CO_2_ supply at the carboxylation action site, which increases the *δ*^13^C value of plants, indicating that plants accumulate heavy carbon isotopes and deplete diazo isotopes under environmental stress conditions [42]. Water conditions are the most crucial factor influencing the nitrogen availability of plants and controlling the nitrogen cycle in ecosystems. Niu et al. [22] found that leaf *δ*^13^C was positively correlated with AI and SS, while leaf *δ*^15^N was negatively correlated with AI and SS. The conclusion of this study is consistent with previous research. With the increase in drought severity (AI) and salt stress (SS), *δ*^13^C, which represents water use efficiency, increases, while *δ*^15^N, which represents nitrogen use, significantly decreases. Among the leaf functional traits related to comprehensive utilization of water and nitrogen, Ci, E, SLA, and LTK decrease, while Pro increases.

In the perennially low-temperature area of the Qinghai–Tibet Plateau, water use efficiency is highly sensitive to temperature. Higher temperatures may cause plants to reduce water loss by regulating stomatal opening, thereby lowering the transpiration rate to minimize excessive water loss. This leads to a sharp decline in the CO_2_ exchange rate of leaves as the temperature rises. Plants respond to drought by accumulating proline. This self-regulating mechanism helps to enhance the water use efficiency of plants in arid environments (leaf *δ*^13^C) [47]. As the temperature rises, it will accelerate the evaporation of water in the soil, leading to a decline in the ability of plant roots to absorb water and nutrients, and thereby affecting the potassium content in leaves [48]. In this study, with the increase in GST, the transpiration rate of plants and the intercellular carbon dioxide concentration decreased significantly, while the water use efficiency (*δ*^13^C) and proline content of plants continued to increase. Nitrogen is an important element for the formation of plant cells and tissues. An increase in total nitrogen in the soil can enhance the growth rate and biomass of plants, thereby increasing chlorophyll content (Chl), strengthening photosynthesis, and raising stomatal conductance (GSW). This helps plants maintain high water content in leaves and mitigate the negative effects of drought [49,50]. In this case, plants may not need to accumulate proline to cope with adversity. Therefore, as STN increases, the growth conditions of plants improve, adverse stress decreases, and the accumulation of Pro also decreases accordingly. The increase in STN can enhance the specific leaf area and leaf nutrient content, thereby strengthening the physiological activity of plants, including increasing stomatal conductance, enhancing photosynthesis and transpiration rates, and thus improving the plant’s ability to obtain resources (leaf *δ*^15^N) [51]. In this study, *δ*^15^N and LTK were positively correlated with STN, while Pro was significantly negatively correlated with STN Ci, E, and SLA generally show an upward trend with the increase in STN.

**Figure 8:**
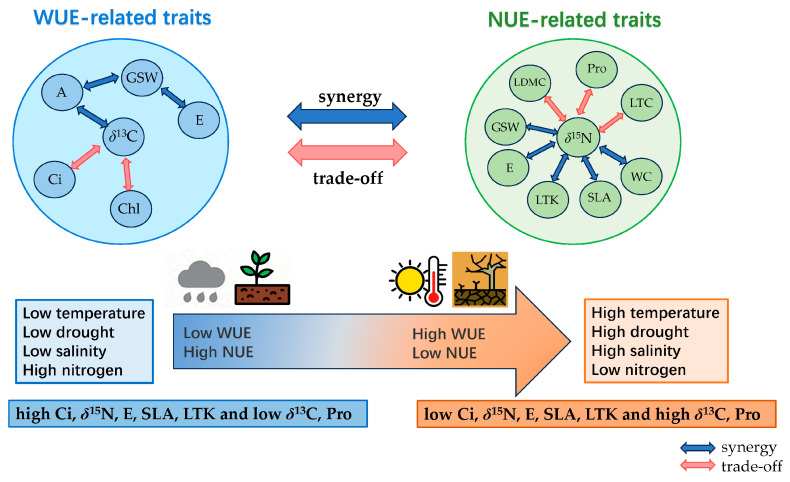
The trade-off or synergistic relationship of plant water and nitrogen utilization efficiency and related functional traits under environmental gradient.

According to the aforementioned analysis, there exists a coupling (trade-off) relationship between the utilization of water and nitrogen. Leaf stable carbon isotope (*δ*^13^C), stable nitrogen isotope (*δ*^15^N), net photosynthetic rate (A), transpiration rate (E), intercellular carbon dioxide concentration (Ci), chlorophyll content (Chl), proline content (Pro), leaf total potassium (LTK), and specific leaf area (SLA) are important plant functional traits that affect the comprehensive utilization of water and nitrogen. Among them, *δ*^13^C, *δ*^15^N, Ci, E, Pro, LTK, and SLA show a variation pattern along the gradient of environmental factors. Plants make a trade-off between *δ*^13^C and Ci, and the synergy between *δ*^15^N and E, SLA, and LTK, as well as the trade-off with Pro, and the trade-off between *δ*^13^C and *δ*^15^N, forming an leaf economic spectrum of water–nitrogen coupling utilization; that is, at the end of high temperature, high drought, high salinity, and low nitrogen, there is a combination of traits reflecting high water use efficiency and low nitrogen use efficiency—low Ci, *δ*^15^N, E, SLA, and LTK and high *δ*^13^C and Pro. At the end of low temperature, low drought, low salinity and high nitrogen, there is a combination of traits reflecting low water use efficiency and high nitrogen use efficiency—high Ci, *δ*^15^N, E, SLA, and LTK and low *δ*^13^C and Pro (Figure 8).

## 4. Materials and Methods

### 4.1. Study Area

**Figure 9:**
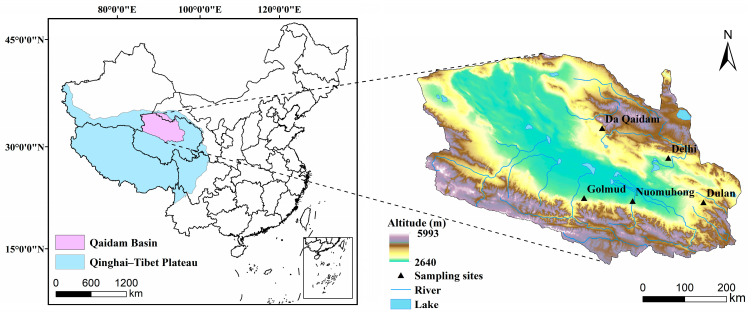
Geographic location of the study area.

The Qaidam Basin is located in the northeastern part of the Qinghai–Tibet Plateau and the northwestern part of Qinghai Province, China, and the basin altitude ranges from 2640 to 5993 m (Figure 9). The Qaidam Basin is dominated by desert ecosystems, in which the main vegetation life forms are desert shrubs (*Ephedra sinica*, *Kalidium foliatum*, *Calligonum mongolicum*, etc.), subshrubs (*Ceratoides latens*, etc.), and micro-subshrubs (*Sympegma regelii, Salsola abrotanoides*, etc.) [52], which account for about 42.6% of the vegetation cover area of the Qaidam Basin [53]. Its growth conditions can better reflect the water and nutrient utilization of high-altitude desert shrubs and their relationship with environmental factors.

### 4.2. Field Survey and Sampling

Field sampling was completed in five sample sites with different aridity degrees in the eastern part of the Qaidam Basin, namely, Golmud, Nuomuhong, Da Qaidam, Delhi, and Dulan (the average precipitation for the years 1990–2020 was 56.2 mm, 68.6 mm, 98.6 mm, 191.1 mm, and 256.5 mm, respectively) (Figure 9). Sample sites were selected in sections with consistent plant community types and relatively flat and open terrain, and three plant community survey sample squares were set in each sample site. Sample squares of different sizes of 5 m × 5 m or 10 m × 10 m were established based on vegetation life forms, and relevant information such as vegetation cover, plant height, species, and elevation were recorded, and plant leaf samples of all species were collected (Table 1). Three soil profiles per plot were randomly selected, and samples were obtained at depths of 0–10 cm, 10–30 cm, and 30–50 cm through mechanical stratification. Five sample sites were sampled every two weeks from June to September 2010, and a total of 396 plant leaf samples from 8 dominant species were collected for the determination of *δ*^13^C and *δ*^15^N. In July to August 2021, a total of 72 plant samples of 9 dominant species and 45 soil samples were collected from the same 5 sites for the determination of other physiological and ecological indicators of plant leaves, except *δ*^13^C and *δ*^15^N, and soil physicochemical indicators.

### 4.3. Experimental Method

Leaf stable carbon isotopes (*δ*^13^C) and stable nitrogen isotopes (*δ*^15^N) were determined from dried plant leaf samples by a high-precision isotope mass spectrometer (Finnigan MAT-253, Thermo Fisher Scientific, Bremen, Germany), and the standard error for repeated measurements was ±0.2‰, which was calculated by the following formula:

(1) $$\delta^{13}C\left(‰\right)=\left(\frac{R_{\mathrm{sample}}}{R_{\mathrm{PDB}}}-1\right)\times 1000$$

(2) $$\delta^{15}N\left(‰\right)=\left(\frac{R_{\mathrm{sample}}}{R_{\mathrm{air}}}-1\right)\times 1000$$

where R_sample_, R_PDB_, and R_air_ are the ratio of ^13^C/^12^C or ^15^N/^14^N of leaf samples, IGS V-PDB and N_2_ in air, respectively, R_PDB_ = 0.0112372 and R_air_ = 0.0036765.

Net photosynthetic rate (A), transpiration rate (E), intercellular carbon dioxide concentration (Ci), and stomatal conductance (GSW) of dominant species of the plant community were recorded using a portable photosynthesis meter (LI-6800, LI-COR Biosciences, Lincoln, NE, USA) from 9:00 to 11:00 a.m. on a sunny, cloudless, or less cloudy day. Parameters: A built-in light source (light intensity 1500 µmol·m^−2^·s^−1^) and a built-in CO_2_ cylinder provided the gas source, and the CO_2_ concentration was 400 µmol·mol^−1^, the relative humidity was 55%, and the fixed flow rate was 500 μmol·s^−1^. The area of fresh leaves was measured by using millimeter paper, and the fresh weight was measured by using a one-thousandth-of-a-milliliter balance, and then the leaves were dried in an oven at 70 °C for 48 h to a constant weight and then weighed at their dry weight, and the leaf’s specific leaf area (SLA) and leaf dry matter content (LDMC) were calculated. The chlorophyll (Chl) content was determined by spectrophotometer [54], and the proline content (Pro) was extracted by sulfosalicylic acid and determined by the ninhydrin colorimetric method [55]. The leaf total phosphorus (LTP) was determined using H_2_SO_4_—H_2_O_2_ decoction and the vanadium–molybdenum yellow colorimetric method (UV-2600i, Shimadzu, Kyoto, Japan) [6]. The leaf total potassium (LTK) was determined using H_2_SO_4_—H_2_O_2_ decoction and the flame photometric method (AA240FS, Varian, Palo Alto, CA, USA) [56], and the plant total nitrogen (LTN) and organic carbon (LTC) contents were determined by an elemental analyzer (EA3000, Euro Vector, Pavia, Italy) [57]. The community-weighted mean value of leaf functional traits was calculated by the sum of the products of the relative coverage weights and the trait values of that species [58].

The soil total phosphorus (STP) was determined by molten the alkali-molybdenum antimony colorimetric method (UV-2600i, Shimadzu, Kyoto, Japan) [59], and the soil total potassium (STK) was determined by the molten alkali-flame photometer method (AA240FS, Varian, Palo Alto, CA, USA) [59]. The soil total nitrogen (STN) and organic carbon (SOC) contents were determined by an elemental analyzer (EA3000, Euro Vector, Pavia, Italy) [60]. The soil water content (SWC) was determined by the drying method. Air-dried soil was mixed with deionized water in a ratio of a 1:5 mixture to form a suspension and then leached. Soil pH was determined, and its conductivity value was measured by a conductivity meter (DDS-11A, Leici, Shanghai, China), which was used to characterize the soil’s soluble total salt (SS) content [52].

### 4.4. Digital Elevation Model and Climate Data Acquisition

Digital elevation model (DEM) data with a spatial resolution of 30 m were obtained from the Geospatial Data Cloud (http://www.gscloud.cn/, accessed on 1 September 2023). Climatic variables, including growing season temperature (GST), growing season precipitation (GSP), mean annual precipitation (MAP), and relative humidity (RH), were acquired from meteorological stations near the sampling sites (including Mangya, Lenghu, Xiaozaohuo, Da Qaidam, Delhi, Golmud, Nuomuhong, Dulan), using the “China National Ground Meteorological Station Basic Meteorological Elements Daily Values Dataset (V3.0)”, provided by the Meteorological Information Center of the China Meteorological Administration (https://data.cma.cn/, accessed on 1 October 2023), and the data of the basic meteorological elements of the national ground meteorological stations were selected for the period of 1990–2020’s month-by-month precipitation and temperature data. The potential evapotranspiration (PET) data were extracted from the “Potential Evapotranspiration in Qaidam Basin 1901–2020”, provided by Chengdu Institute of Mountain Hazards and Environment, Chinese Academy of Sciences, at a resolution of 1 km, and the data were extracted from the sampling sites during the period of 1990–2020. The annual aridity index (AI) is the ratio of potential evapotranspiration (PET) to annual precipitation (MAP). The saturated water vapor pressure difference (VPD) was estimated based on Equation (3):

(3) $$\mathrm{VPD}=0.6108\times \exp\left(\frac{17.27T_{a}}{T_{a}+237.3}\right)\times \left(\frac{1-\mathrm{RH}}{100}\right)$$

where T_a_ denotes air temperature (°C); RH denotes relative humidity (%).

### 4.5. Data Analysis Method

Network analysis of plant functional traits was conducted using JASP software (JASP 0.18.1.0, University of Amsterdam, Amsterdam, The Netherlands), and variables with strong collinearity were excluded. Network nodes represent leaf traits. The edge represents the correlation between two nodes, and the thickness of the line indicates the strength of the correlation. By bivariate regression analysis, the relationship between *δ*^13^C and *δ*^15^N, and the relationship between *δ*^13^C and *δ*^15^N and leaf functional traits (A, Ci, Chl, E, GSW, SLA, WC, Pro, LDMC, LTC, LTK) were examined, and the changes in main leaf functional traits along the environmental gradient were further analyzed. Redundancy analysis (RDA) and hierarchical segmentation were employed to examine the influence of leaf functional traits on water and nitrogen use efficiency, and the contribution degree of each influencing factor was ranked. Data analysis and mapping were carried out in R version 4.3.2 (R Core Team, Vienna, Austria) and Origin version 2022 (OriginLab Corporation, Northampton, MA, USA) software. The relationship analysis of *δ*^13^C and *δ*^15^N at the community scale was conducted using the community-weighted mean value, while other analyses all adopted plant functional trait data at the species scale.

## 5. Conclusions

In conclusion, in this study, the leaf functional traits of desert shrubs in the Qaidam Basin related to resource utilization can be divided into two groups: those related to water utilization and those related to nitrogen utilization. Among the water utilization-related traits, *δ*^13^C exhibits synergistic relationships with A and trade-offs with Ci and Chl. Among the nitrogen utilization-related traits, *δ*^15^N shows synergistic relationships with E, GSW, SLA, WC, and LTK, and trade-offs with Pro and LDMC. The functional traits of plant leaves that affect the comprehensive utilization of water and nitrogen include *δ*^13^C, Ci, A, Chl, *δ*^15^N, E, Pro, LTK, and SLA. Among them, *δ*^13^C, Ci, *δ*^15^N, E, Pro, LTK, and SLA show regular changes along the gradients of GST, AI, SS, and STN. The combined characteristics of leaf functional traits of typical desert shrubs in the Qaidam Basin form an economic spectrum of water and nitrogen utilization along the environmental gradient, and there is a trade-off relationship between water and nitrogen utilization. This way of optimizing resource utilization indicates that there are different combinations of water and nutrient traits in the desert ecosystem. Plants adopt high WUE and low NUE to cope with high temperature, high drought, high salt, and low nitrogen environments, as well as high NUE and low WUE to cope with low temperature, low drought, low salt, and high nitrogen environments. This study reveals the physiological and ecological mechanisms by which temperate high-altitude desert shrubs adapt to arid environments from a functional ecology perspective. Furthermore, future research could delve into comparing changes in plant leaf functional traits across different aridity gradients or environmental factor gradients, and conduct comparative studies on desert shrubs with diverse functional types. This would enable a more comprehensive understanding of the response mechanisms in temperate high-altitude desert ecosystems, providing a scientific basis for predicting the evolutionary trends of vegetation patterns in temperate high-altitude desert regions under climate change.

## Figures and Tables

**Table 1:** Species composition, location, climate, and soil properties of plant communities in the sample sites.

Sites	Community Types (Total Community Cover: %)	Long (°E)	Lat (°N)	Alt (m)	GST (°C)	GSP (mm)	Aridity Index	Soil Soluble Salts (EC25 ms/cm)
Golmud	*C.m.* + *S.r.* + *E.s.* + *C.l.* (17%)	95.09	36.35	2880	15.02	45.97	28.98	2.79
Nuomuhong	*E.s.* + *C.m.* + *N.t.* + *C.l.* + *T.c.* (15%)	96.41	36.38	2857	14.84	55.96	23.29	1.87
Da Qaidam	*C.l.* + *S.r.* + *S.a.* (10%)	95.4	37.86	3315	10.47	81.24	13.76	0.54
Delhi	*K.f.* + *S.r.* (25%)	97.28	37.35	2972	14.43	150.29	8.17	0.11
Dulan	*K.f.* + *S.a.* (55%)	98.33	36.47	3323	10.89	200.71	4.92	0.16

*C.m.*: *Calligonum mongolicum*; *S.r.*: *Sympegma regelii*; *E.s.*: *Ephedra sinica*; *C.l.*: *Ceratoides latens*; *N.t.*: *Nitraria tangutorum*; *T.c.*: *Tamarix chinensis*; *S.a.*: *Salsola abrotanoides*; *K.f.*: *Kalidium foliatum*. Climatic factors are the sum precipitation and average temperature during the plant growing season (GSP and GST, respectively) and the habitat aridity index (AI). The habitat aridity index is defined as the ratio of PET/MAP.

## Data Availability

The data presented in this study are available on request from the corresponding author. The data are not publicly available due to privacy and ethical restrictions.

## References

[1] Wang Y., Lü Y., Lü D., Yin L., Wang X. (2024). Climate change and its ecological risks are spatially heterogeneous in high-altitude region: The case of Qinghai-Tibet plateau. CATENA.

[2] Yao J., Liu H., Huang J., Gao Z., Wang G., Li D., Yu H., Chen X. (2020). Accelerated dryland expansion regulates future variability in dryland gross primary production. Nat. Commun..

[3] Zhao L., Chen H., Chen B., Wang Y., Sun H. (2022). Drought Shapes Photosynthetic Production Traits and Water Use Traits along with Their Relationships with Leaves of Typical Desert Shrubs in Qaidam. Forests.

[4] Zhao D., Wei M., Wang X., Aqeel M., Ran J., Deng J. (2024). Morpho-physiological adaptations to drought stress in nitrogen-fixing and non-nitrogen-fixing plants. Front. Ecol. Evol..

[5] Liu W., Zheng L., Qi D. (2020). Variation in leaf traits at different altitudes reflects the adaptive strategy of plants to environmental changes. Ecol. Evol..

[6] Fan B., Westerband A.C., Wright I.J., Gao P., Ding N., Ai D., Tian T., Zhao X., Sun K. (2023). Shifts in plant resource use strategies across climate and soil gradients in dryland steppe communities. Plant Soil.

[7] Ding Y., Nie Y., Chen H., Wang K., Querejeta J.I. (2021). Water uptake depth is coordinated with leaf water potential, water-use efficiency and drought vulnerability in karst vegetation. New Phytol..

[8] Gauthier P.P.G., Lamothe M., MahÉ A., Molero G., NoguÉS S., Hodges M., Tcherkez G. (2013). Metabolic origin of δ^15^N values in nitrogenous compounds from Brassica napus L. leaves. Plant Cell Environ..

[9] Robinson D. (2001). δ^15^N as an integrator of the nitrogen cycle. Trends Ecol. Evol..

[10] Liu G., Wang Q., Chen J., Yan G., Wang H., Xing Y. (2024). Natural δ^13^C and δ^15^N Abundance of Plants and Soils Under Long-term N Addition in a Temperate Secondary Forest. J. Soil Sci. Plant Nutr..

[11] Hu J., Ma W., Wang Z. (2023). Effects of nitrogen addition and drought on the relationship between nitrogen- and water-use efficiency in a temperate grassland. Ecol. Process..

[12] Conesa H.M., Párraga-Aguado I.M., Jiménez F.J., Querejeta J.-I. (2024). Evaluation of the trade-off between water use efficiency and nutrient use efficiency in two semiarid coniferous tree species growing on an organic amended metalliferous mine tailing substrate. Sci. Total Environ..

[13] Lu S., Chen Y., Sardans J., Peñuelas J. (2024). Water and nutrient use efficiency of three tree species in monoculture and mixed stands and potential drivers in the Loess Hilly Region, China. Plant Soil.

[14] Zhao L., Xiao H., Cheng G., Liu X., Yang Q., Yin L., Li C. (2010). Correlation between δ^13^C and δ^15^N in C_4_ and C_3_ plants of natural and artificial sand-binding microhabitats in the Tengger Desert of China. Ecol. Inform..

[15] Gong X.Y., Chen Q., Lin S., Brueck H., Dittert K., Taube F., Schnyder H. (2011). Tradeoffs between nitrogen- and water-use efficiency in dominant species of the semiarid steppe of Inner Mongolia. Plant Soil.

[16] Huang Z., Liu B., Davis M., Sardans J., Peñuelas J., Billings S. (2016). Long-term nitrogen deposition linked to reduced water use efficiency in forests with low phosphorus availability. New Phytol..

[17] Cheng L., Zhang B., Zhang H., Li J. (2022). Biomass, Carbon and Nitrogen Partitioning and Water Use Efficiency Differences of Five Types of Alpine Grasslands in the Northern Tibetan Plateau. Int. J. Environ. Res. Public Health.

[18] Joshi R.K., Gupta R., Mishra A., Garkoti S.C. (2024). Seasonal variations of leaf ecophysiological traits and strategies of co-occurring evergreen and deciduous trees in white oak forest in the central Himalaya. Environ. Monit. Assess..

[19] Su B., Shangguan Z. (2020). Patterns and driving factors of water and nitrogen use efficiency in Robinia pseudoacacia L. on the Loess Plateau in China. CATENA.

[20] Crous K.Y., O’Sullivan O.S., Zaragoza-Castells J., Bloomfield K.J., Negrini A.C.A., Meir P., Turnbull M.H., Griffin K.L., Atkin O.K. (2017). Nitrogen and phosphorus availabilities interact to modulate leaf trait scaling relationships across six plant functional types in a controlled-environment study. New Phytol..

[21] Sun L., Li J., Qu L., Wang X., Sang C., Wang J., Sun M., Wanek W., Moorhead D.L., Bai E. (2023). Phosphorus limitation reduces microbial nitrogen use efficiency by increasing extracellular enzyme investments. Geoderma.

[22] Niu W., Chen H., Wu J. (2021). Soil Moisture and Soluble Salt Content Dominate Changes in Foliar δ^13^C and δ^15^N of Desert Communities in the Qaidam Basin, Qinghai-Tibetan Plateau. Front. Plant Sci..

[23] Joshi R.K., Mishra A., Gupta R., Garkoti S.C. (2024). Leaf and tree age-related changes in leaf ecophysiological traits, nutrient, and adaptive strategies of Alnus nepalensis in the central Himalaya. J. Biosci..

[24] Ye X., Bu W., Hu X., Liu B., Liang K., Chen F. (2023). Species divergence in seedling leaf traits and tree growth response to nitrogen and phosphorus additions in an evergreen broadleaved forest of subtropical China. J. For. Res..

[25] Hu C.-C., Liu X.-Y., Driscoll A.W., Kuang Y.-W., Brookshire E.N.J., Lü X.-T., Chen C.-J., Song W., Mao R., Liu C.-Q. (2024). Global distribution and drivers of relative contributions among soil nitrogen sources to terrestrial plants. Nat. Commun..

[26] Pan S., Wang X., Yan Z., Wu J., Guo L., Peng Z., Wu Y., Li J., Wang B., Su Y. (2024). Leaf stomatal configuration and photosynthetic traits jointly affect leaf water use efficiency in forests along climate gradients. New Phytol..

[27] Rehaman A., Khan S., Rawat B., Gaira K.S., Asgher M., Semwal P., Tripathi V. (2025). Mechanistic Insights into Plant Drought Tolerance: A Multi-level Perspective. J. Crop Health.

[28] Li P., Zhu Y., Song X., Song F. (2020). Negative effects of long-term moderate salinity and short-term drought stress on the photosynthetic performance of Hybrid Pennisetum. Plant Physiol. Biochem..

[29] Wright I.J., Reich P.B., Westoby M., Ackerly D.D., Baruch Z., Bongers F., Cavender-Bares J., Chapin T., Cornelissen J.H.C., Diemer M. (2004). The worldwide leaf economics spectrum. Nature.

[30] Ma J., Chen F., Xia D., Sun H., Duan Z., Wang G. (2008). Correlations between leaf δ^13^C and physiological parameters of desert plant Reaumuria soongorica. Chin. J. Appl. Ecol..

[31] Tian Z. (2021). Relationship of Main Nutrient Elements in Soil and Vegetation in Qaidam Basin. Master’s Thesis.

[32] Wellstein C., Poschlod P., Gohlke A., Chelli S., Campetella G., Rosbakh S., Canullo R., Kreyling J., Jentsch A., Beierkuhnlein C. (2017). Effects of extreme drought on specific leaf area of grassland species: A meta-analysis of experimental studies in temperate and sub-Mediterranean systems. Glob. Change Biol..

[33] Chen S., Bai Y., Zhang L., Han X. (2005). Comparing physiological responses of two dominant grass species to nitrogen addition in Xilin River Basin of China. Environ. Exp. Bot..

[34] Swap R.J., Aranibar J.N., Dowty P.R., Gilhooly W.P., Macko S.A. (2004). Natural abundance of ^13^C and ^15^N in C_3_ and C_4_ vegetation of southern Africa: Patterns and implications. Glob. Change Biol..

[35] Reich P.B., Ellsworth D.S., Walters M.B., Vose J.M., Gresham C., Volin J.C., Bowman W.D. (1999). Generality of leaf trait relationships: A test across six biomes. Ecology.

[36] Lin Y., Grace J., Zhao W., Dong Y., Zhang X., Zhou L., Fei X., Jin Y., Li J., Nizami S.M. (2018). Water-use efficiency and its relationship with environmental and biological factors in a rubber plantation. J. Hydrol..

[37] Farquhar G.D., Ehleringer J.R., Hubick K.T. (1989). Carbon Isotope Discrimination and Photosynthesis. Annu. Rev. Plant Biol..

[38] Liu Z., Zhao M., Zhang H., Ren T., Liu C., He N. (2023). Divergent response and adaptation of specific leaf area to environmental change at different spatio-temporal scales jointly improve plant survival. Glob. Change Biol..

[39] Akram M.A., Zhang Y., Wang X., Shrestha N., Malik K., Khan I., Ma W., Sun Y., Li F., Ran J. (2022). Phylogenetic independence in the variations in leaf functional traits among different plant life forms in an arid environment. J. Plant Physiol..

[40] Luo Y., Peng Q., He M., Zhang M., Liu Y., Gong Y., Eziz A., Li K., Han W. (2020). N, P and K stoichiometry and resorption efficiency of nine dominant shrub species in the deserts of Xinjiang, China. Ecol. Res..

[41] Wu J., Song M., Ma W., Zhang X., Shen Z., Tarolli P., Wurst S., Shi P., Ratzmann G., Feng Y. (2019). Plant and soil’s δ^15^N are regulated by climate, soil nutrients, and species diversity in alpine grasslands on the northern Tibetan Plateau. Agric. Ecosyst. Environ..

[42] Min X., Ma J., Bahjayrarl T., Zang Y. (2017). Effects of Water and Salinity Stress on Carbon and Nitrogen Isotopic Compositions in Leaves of Tamarix elongata and *Haloxylon ammodendron*. Arid Zone Res..

[43] Zhang X., Duan J., Ji Y., Liu W., Gao J. (2024). Leaf nutrient traits exhibit greater environmental plasticity compared to resource utilization traits along an elevational gradient. Front. Plant Sci..

[44] Donnelly R.C., Nippert J.B., Wedel E.R., Ferguson C.J. (2024). Grass leaf structural and stomatal trait responses to climate gradients assessed over the 20th century and across the Great Plains, USA. AoB Plants.

[45] Konings A.G., Williams A.P., Gentine P. (2017). Sensitivity of grassland productivity to aridity controlled by stomatal and xylem regulation. Nat. Geosci..

[46] Dong X., Li Y., Xin Z., Liu M., Hao Y., Liu D., Chen X., Zhang Z. (2019). Variation in leaf traits and leaf δ^13^C and δ^15^N content in *Nitraria tangutorum* along precipitation gradient. Acta Ecol. Sin..

[47] Driscoll A.W., Bitter N.Q., Sandquist D.R., Ehleringer J.R. (2020). Multidecadal records of intrinsic water-use efficiency in the desert shrub Encelia farinosa reveal strong responses to climate change. Proc. Natl. Acad. Sci. USA.

[48] Cakmak I., Rengel Z. (2024). Humboldt Review: Potassium may mitigate drought stress by increasing stem carbohydrates and their mobilization into grains. J. Plant Physiol..

[49] Sun M., Tian K., Zhang Y., Wang H., Guan D., Yue H. (2017). Research on leaf functional traits and their environmental adaptation. Plant Sci. J..

[50] Wang D., Duan H., Zhao Y., Qiu W., Liu X., Wu J., Huang G., Liu W. (2025). Antecedent moderate nitrogen fertilization alleviated the effects of drought on growth and leaf photosynthesis of *Schima Superba* seedlings. BMC Plant Biol..

[51] Hu Y., Yao X., Liu Y. (2015). Specific leaf area and its influencing factors of forests at different succession stages in Changbai Mountains. Acta Ecol. Sin..

[52] Zhao L. (2023). Water-Carbon Balance Mechanism of Typical Desert Shrubs in Eastern Qaidam Based on Leaf Functional Traits. Master’s Thesis.

[53] Chen B., Chen H., Li M., Fiedler S., Mărgărint M.C., Nowak A., Wesche K., Tietjen B., Wu J. (2022). Climate Sensitivity of the Arid Scrublands on the Tibetan Plateau Mediated by Plant Nutrient Traits and Soil Nutrient Availability. Remote Sens..

[54] Lichtenthaler H.K., Wellburn A.R. (1983). Determinations of total carotenoids and chlorophylls a and b of leaf extracts in different solvents. Biochem. Soc. Trans..

[55] Singh R., Saffeullah P., Umar S., Abass S., Ahmad S., Iqbal N. (2024). Comparing the individual and combined effects of nano zinc and conventional zinc fertilization on growth, yield, phytochemical properties, antioxidant activity, and secoisolariciresinol diglucoside content in linseed. Plant Nano Biol..

[56] Heydarzadeh S., Tobeh A., Jahanbakhsh S., Farzaneh S., Vitale E., Arena C. (2024). The Application of Stress Modifiers as an Eco-Friendly Approach to Alleviate the Water Scarcity in Ajwain (*Carum copticum* L.) Plants. Plants.

[57] Liu M.-C., Dong T.-F., Feng W.-W., Qu B., Kong D.-L., Kleunen M.v., Feng Y.-L. (2022). Leaf trait differences between 97 pairs of invasive and native plants across China: Effects of identities of both the invasive and native species. NeoBiota.

[58] Chen J., Song N., Wang X., Meng C., Zhang Y., Chen L., Wang Q., Lv H., Wu X., Yu D. (2024). Precipitation and plant community-weighted mean traits determine total transpirable soil water in a desert grassland. Ecol. Indic..

[59] Chen S., Sun Y., Yang D., Yang S., Liang T., Tan H. (2022). Using moss as a bio-indicator to evaluate soil quality in litchi orchard. PLoS ONE.

[60] Zhang Z., Guo Y., Ye S., Wang S. (2024). Response of Soil Microbial Communities and Nutrient Stoichiometry to Stand Age in Chinese Fir Plantations: Insights at the Aggregate Scale. J. Soil Sci. Plant Nutr..

